# Oral manifestations in HIV-positive individuals under highly active antiretroviral therapy: a systematic review and meta-analysis of prevalence data

**DOI:** 10.1186/s12903-026-08182-0

**Published:** 2026-03-31

**Authors:** Thais Suzigan Dagnoni, Luiz Renato Paranhos, Vinícius Lima de Almeida, Walbert de Andrade Vieira, João Botelho, Verena Paula Stern Netto, Micena Roberta Miranda Alves e Silva, Ademir Franco, Rui Barbosa de Brito Júnior

**Affiliations:** 1 Post-Graduation Program in Dentistry, School of Dentistry, Faculdade São Leopoldo Mandic (SLM), Campinas, SP Brazil; 2 Department of Orthodontics, School of Dentistry, Universidade Federal de Uberlândia (UFU), Campus Umuarama, Av. Pará, nº 1720, Block 2G, Room 2, Uberlândia, MG 38405-320 Brazil; 3 Post-Graduation Program in Dentistry, School of Dentistry, Universidade Federal de Uberlândia (UFU), Uberlândia, MG Brazil; 4 School of Dentistry, Centro Universitário das Faculdades Associadas de Ensino (UNIFAE), São João da Boa Vista, SP Brazil; 5 Egas Moniz School of Health & Science, Egas Moniz Center for Interdisciplinary Research, Almada, 2829-511 Portugal; 6 Institute of Biological Sciences, Universidade Federal de Minas Gerais (UFMG), Belo Horizonte, MG Brazil; 7 Division of Forensic Dentistry, Faculdade São Leopoldo Mandic (SLM), Campinas, SP Brazil; 8 Department of Molecular Biology, School of Dentistry, Faculdade São Leopoldo Mandic (SLM), Campinas, SP Brazil

**Keywords:** Antiretroviral therapy, HAART, Highly active antiretroviral therapy, HIV

## Abstract

This study aims to analytically explore the prevalence of oral manifestations in HIV-positive patients who are receiving or are not receiving highly active antiretroviral therapy (HAART). Electronic search was conducted at multiple databases (PubMed, Embase, LILACS, SciELO, Scopus and Web of Science) and gray literature was also assessed (ProQuest and EASY databases). JBI Critical Appraisal Tools were used to establish methodological quality. Data from each study was combined in a meta-analysis of proportions using an inverse variance method, Freeman-Tukey double arcsine transformation, and Clopper-Pearson confidence interval. Quality of evidence was determined via Grading of Recommendations, Assessment, Development, and Evaluation (GRADE) approach. The search yielded 25 eligible articles, with quantitative synthesis from 13. Only four studies had a low risk of bias. The meta-analysis revealed a prevalence of oral manifestations in 42% (95% CI: 29% – 56%, I^2^ = 97%) of patients receiving HAART. Patients undergoing HAART were less likely to present oral manifestations (OR: 0.51; 95% CI: 0.27–0.94) than those in the non-HAART group, with a very low certainty of evidence. Although patients receiving HAART are less likely to present with oral manifestations, primary studies must be performed with a standardized methodology to guarantee results with a higher certainty of evidence.

**Supplementary Information:**

The online version contains supplementary material available at 10.1186/s12903-026-08182-0.

## Background

Highly active antiretroviral therapy (HAART) has revolutionized human immunodeficiency virus (HIV) treatment, dramatically reducing mortality and transforming a fatal disease into a controllable chronic condition [1]. However, its effects go beyond controlling the viral load and directly impact the immune system and clinical manifestations in various parts of the body, including the oral cavity, which often reflects the patient's immune status [2].

Before the introduction of HAART, the oral manifestations of candidiasis, hairy leukoplakia, Kaposi's sarcoma, and necrotizing periodontal diseases were widely prevalent, affecting up to 80% of untreated patients [3]. These findings not only indicated severe immunosuppression but also the compromise of basic functions, such as chewing and speech, severely impacting the quality of life of individuals [4]. Following the advent of HAART, the prevalence of these conditions decreased significantly [5]. For instance, the occurrence of oral candidiasis, one of the most common fungal infections, has reduced to less than 30% in treated populations, and hairy leukoplakia, once prevalent in patients with low CD4 + counts, now occurs in less than 5% of cases [6, 7].

However, the introduction of HAART has promoted new challenges, such as the immune reconstitution inflammatory syndrome. This syndrome affects up to 20% of patients, and it may trigger oral manifestations, such as the reactivation of herpes simplex and exacerbation of periodontal diseases [8, 9]. Although less frequent, these conditions require clinical attention to prevent complications and ensure adherence to the antiretroviral treatment [10].

Additionally, new evidence highlights the role of inflammatory cytokines, such as IL-6 and TNF-α, in mediating oral inflammatory responses during HAART [11]. These markers help understand the underlying mechanisms of oral manifestations and guide clinical management strategies [12]. Moreover, adverse effects associated with the therapy, such as drug-induced oral hyperpigmentation, remain significant challenges for patients under prolonged treatment [13].

Despite these adversities, the oral health benefits of HAART are evident [11]. Treated patients present significant improvements in quality of life, with reduced pain and enhanced aesthetics and oral functionality. These achievements underscore the relevance of integrating dental care into HIV management strategies, enabling an interdisciplinary approach [10]. Thus, this study analytically assesses the prevalence of oral manifestations in HIV-positive patients receiving or not receiving HAART, highlighting the significance of collaboration among health specialists to maximize the therapeutic benefits and minimize the challenges associated with treatment.

## Methods

### Protocol and registration

This systematic review was reported following the guidelines of the Preferred Reporting Items for Systematic Reviews and Meta-Analyses (PRISMA) [14], registered in the database of the Prospective International Registry of Systematic Reviews (PROSPERO) under number CRD42024628397 (https://www.crd.york.ac.uk/PROSPERO/), and reported in accordance with the COSMOS-E guidelines [15] and the Joanna Briggs Institute Manual (JBI) [16].

### Eligibility criteria

This systematic review aims to answer the following question: "Does highly active antiretroviral therapy influence the prevalence of oral manifestations in HIV-positive patients?" The question was based on the CoCoPop (Condition, Context, and Population) framework, following JBI recommendations [16].

The primary outcome included observational studies comparing the prevalence of oral lesions in HIV-positive adult patients receiving and not receiving HAART. Secondary outcomes comprised observational studies whose prevalence of oral manifestations was determined only in HIV-positive patients before and after HAART and whose samples included only patients receiving HAART. This review excluded observational studies with samples involving the analysis of oral manifestation prevalence only in HIV-positive children, studies with HIV-positive patients under antiretroviral therapy other than HAART, and studies with HIV-positive patients receiving or not receiving HAART and antifungal treatment. There was no restriction on the language or time of publication.

### Information sources

The electronic searches were conducted in February 2025, on the following databases: MedLine (via PubMed), Embase, LILACS (Latin American and Caribbean Literature in Health Sciences), SciELO, as well as Scopus and Web of Science citation databases. The ProQuest and EASY databases partially captured the gray literature. A manual search was conducted using references from eligible articles. To ensure the review remained current, the initial PubMed search strategy was saved in the "My NCBI" service with email alerts configured for monthly updates. All new results were screened monthly by the same reviewers using the original inclusion and exclusion criteria. This continuous updating process was maintained until the final cut-off date of January 2026. These steps were performed to minimize selection biases.

### Search strategy

The Medical Subject Headings (MeSH), Embase Subject Headings (Emtree), and Health Science Descriptors (DeCS) platforms provided the search descriptors. The Boolean operators “AND” and “OR” combined the keywords. The search strategies respected the syntax rules of each database (Table 1).

**Table 1:** Search strategy used in each database

Databases	Search strategies (November 2024)
Main databases	
MEDLINE (via PubMed) http://www.ncbi.nlm.nih.gov/pubmed	#1 “Antiretroviral Therapy” OR “Highly Active” OR “HAART” OR “Highly Active Antiretroviral Therapy” OR “Anti-HIV Agents” OR “Drug Therapy” OR “Combination” OR “Combination Drug Therapies” OR “Combination Antiretroviral Therapy”
	#2 “Oral Manifestations” OR “Mouth Diseases” OR “Oral Health”
	#1 AND #2
Embase https://www.embase.com	#1 'antiretroviral therapy'/exp OR 'antiretroviral therapy' OR 'highly active' OR 'haart'/exp OR 'haart' OR 'highly active antiretroviral therapy'/exp OR 'highly active antiretroviral therapy' OR 'anti-hiv agents'/exp OR 'anti-hiv agents' OR 'drug therapy'/exp OR 'drug therapy' OR 'combination' OR 'combination drug therapies' OR 'combination antiretroviral therapy'/exp OR 'combination antiretroviral therapy'
	#2 'oral manifestations' OR 'mouth diseases' OR 'oral health'
	#1 AND #2
LILACS http://pesquisa.bvsalud.org/	"Highly Active Antiretroviral THERAPY" AND "Oral Manifestations" AND db:("lilacs" OR "BBO") AND instance:"lilacsplus"
SciELO https://scielo.org/	“Highly Active Antiretroviral Therapy” AND “Oral Manifestations”
	Citation databases
Scopus http://www.scopus.com/	#1 TITLE-ABS-KEY ("Antiretroviral Therapy" OR "Highly Active" OR "HAART" OR "Highly Active Antiretroviral Therapy" OR "Anti-HIV Agents" OR "Drug Therapy" OR "Combination" OR "Combination Drug Therapies" OR "Combination Antiretroviral Therapy")
	#2 TITLE-ABS-KEY ("Oral Manifestations" OR "Mouth Diseases" OR "Oral Health")
	#1 AND #2
Web of Science http://apps.webofknowledge.com/	#1 ALL = (“Antiretroviral Therapy” OR “Highly Active” OR “HAART” OR “Highly Active Antiretroviral Therapy” OR “Anti-HIV Agents” OR “Drug Therapy” OR “Combination” OR “Combination Drug Therapies” OR “Combination Antiretroviral Therapy”)
	#2 ALL = (“Oral Manifestations” OR “Mouth Diseases” OR “Oral Health”)
	#1 AND #2
Gray Literature	
EASY https://easy.dans.knaw.nl/	(“Highly Active Antiretroviral Therapy” OR “HAART”) AND (“Oral Manifestations”)
ProQuest https://www.proquest.com	(“Highly Active Antiretroviral Therapy” OR “HAART”) AND (“Oral Manifestations”)

### Selection process

Primary database findings were exported to EndNote Web™ software (Clarivate™ Analytics, Philadelphia, USA) for the automatic removal of duplicates, and the remainder were excluded manually. The remaining records were exported to Rayyan QCRI (Qatar Computing Research Institute, Doha, Qatar) [17] for the selection and evaluation of titles and abstracts. The gray literature was analyzed manually, simultaneously, and in detail, and exported to Microsoft Word™ 2010 (Microsoft™ Ltd., Washington, USA).

Before study selection, two reviewers (TSD and VLA) performed a calibration exercise in which they discussed the eligibility criteria and applied them to a sample of 20% of retrieved studies to determine inter-examiner agreement. Selection started after achieving an adequate level of agreement (Kappa ≥ 0.81) and occurred in two phases.

In the first phase, two eligibility reviewers (TSD and VLA) methodically analyzed the titles and abstracts of the studies independently. A third examiner (LRP) investigated and resolved disagreements between the reviewers. Titles and abstracts unrelated to the topic were eliminated at this stage, respecting the eligibility criteria. In the second phase, the full texts of the preliminary eligible studies were obtained and evaluated. If the full texts were not found, a bibliographic request was made to the library database (COMUT), and an email was sent to the corresponding authors to retrieve the texts. If the full texts were published in languages other than Portuguese, Spanish, or English, they were translated using the Google Translate tool to prevent selection biases in publication language.

### Data collection process

Two calibrated reviewers (TSD and VLA) independently and blindly extracted the data from eligible studies. A third reviewer (LRP) analyzed conflicts in cases of disagreement about data extraction.

The following data were extracted from the articles: author, year, country, study type, sample, habits/risk factors associated with HIV, the portion of the sample receiving HAART, time under HAART, CD4 + count, viral load, and the prevalence of oral lesions in HIV-positive patients receiving or not receiving HAART. The study did not consider data from patients undergoing only antiretroviral monotherapy.

### Risk of bias assessment

Two reviewers assessed the individual risk of bias of the eligible studies using JBI Critical Appraisal according to their respective study designs [18]. The response options to this tool's questions were “Yes,” “No,” “Uncertain,” or “Not Applicable.” Articles must have a “Yes” answer to all questions to be considered a low risk of bias [16]. A discussion with an expert was required in cases of disagreement between the reviewers.

### Synthesis methods

#### Measures of treatment effect and meta-analysis

The prevalence of oral manifestations in patients receiving HAART was reported as a percentage and with 95% confidence intervals (CIs). The data from each study were combined in a meta-analysis of proportions using the inverse variance method, Freeman-Tukey double arcsine transformation, and Clopper-Pearson confidence interval. A supplementary analysis was conducted to specifically examine the prevalence rates of individual oral manifestations. For a study to be included in this stratified analysis, it was required to report both the prevalence of each manifestation and the total number of participants assessed.

Regarding the dichotomous outcome (oral manifestations in patients receiving HAART vs. not receiving HAART), the intervention effect was expressed using odds ratios (ORs) and 95% CIs. Groups were compared when studies with similar assessments reported the same results. Hence, the studies were combined in meta-analyses using the inverse variance method and random-effects models. The data were summarized with R software for Windows, version 4.5 (R Foundation for Statistical Computing, Vienna, Austria), aided by the “meta” and “metafor” packages.

#### Heterogeneity assessment

This review analyzed the between-study variance using tau-square (τ^2^) statistics and estimated the magnitude of heterogeneity using I-square (I^2^) statistics. Heterogeneity was classified into four categories according to I^2^ statistics [19]:

- 0% to 40% slight heterogeneity;
- 30% to 60% moderate heterogeneity;
- 50% to 90% substantial heterogeneity;
- 75% to 100% considerable heterogeneity.

#### Publication bias

Funnel plots were constructed using the Freeman–Tukey double arcsine transformation for effect sizes and the inverse square root of the sample size (1/√n) on the vertical axis. This approach was adopted because the standard error of proportions depends on the magnitude of the proportion, which may distort the visual assessment of publication bias.

#### Certainty of evidence

Two reviewers determined the quality of evidence using the Grading of Recommendations, Assessment, Development, and Evaluation (GRADE) working group approach [20]. This assessment was based on study design, risk of bias, inconsistency, indirect evidence, imprecision, and other considerations. The certainty of evidence was characterized as high, moderate, low, or very low.

## Results

### Study selection

The electronic search identified 22.243 results distributed across six electronic databases and 1216 results in gray literature. After removing duplicates, 12,413 results remained for analysis. A careful reading of titles and abstracts excluded 12,369 results. Forty-four of the remaining studies were searched for inclusion, but one could not be included for a full-text evaluation due to the impossibility of retrieving the document after attempts to contact the authors and searches in bibliographic exchange systems.

**Fig. 1:**
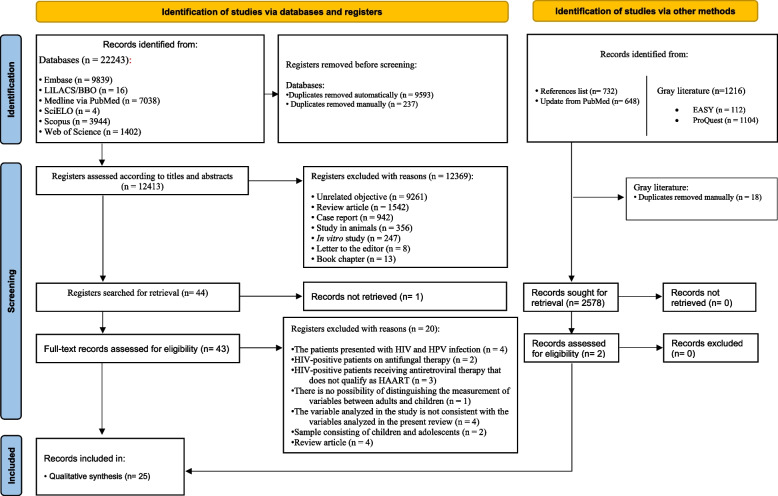
Flowchart of study selection according to the PRISMA statement

After reading the full texts, 20 studies were excluded (Supplementary Table 1 – Online Resource). There was no inclusion of additional studies through grey literature screening or through the monthly PubMed updates up to January 2026. The analysis of the references of the 23 included studies allowed the inclusion of two additional studies. The qualitative synthesis of the study included 25 eligible articles, and the quantitative synthesis had 13 studies (Fig. 1). The Cohen's Kappa coefficient during study selection was 0.806 (95%CI: 0.71; 0.93), indicating an almost perfect level of reliability among reviewers.

### Study characteristics

The articles were published between 2000 and 2023 and conducted in 16 countries. Three studies were published in Spanish [21–23], and the others were published in English [24–45]. As for risk factors for contracting HIV, sexual intercourse and injectable drugs were among the main causal factors, and the male sex represented most of the sample. Among the 25 included studies, 11 did not provide the CD4 + cell count, and only eight revealed the viral load. Table 2 details the general characteristics of the included studies.

**Table 2:** Main characteristics of the included studies

Authors, year, and country	Study type	Sample	Habits/Risk factors	Patients receiving HAART	Time receiving HAART	CD4 + count	Viral load
Tappuni and Fleming, 2001 [41] England	Cross-sectional	284 (229 ♂ 55 ♀)	Sexual intercourse (174) Injectable drugs (14) Other (10)	36	*	< 200 cells/mm^3^ (information regarding 50 patients using any ART) < 200 cells/mm^3^ (information regarding 47 patients not using any ART)	26 patients from the any ART group and 15 patients from the no ART group (< 500 copies/mL) and 18 patients of any ART compared to 37 without ART (> 3,000 copies/mL)
Eyeson et al., 2002 [29] England	Cross-sectional	192 *	Injectable drugs (3)	141	*	*	*
Nicolatou-Galitis et al., 2004 [36] Greece	Cross-sectional	95 (85 ♂ 10 ♀)	*	58	*	0–200 (15 patients without HAART and 13 patients with HAART) 201–500 (11 patients without HAART and 29 patients with HAART) > 500 (11 patients without HAART and 16 patients with HAART)	0–500 (1 patient without HAART and 23 patients with HAART) 501–20,000 (16 patients without HAART and 24 patients with HAART) > 20,000 (37 patients without HAART and 11 patients with HAART)
Kroidl et al., 2005 [32] Germany	Cross-sectional	129	*	129	*	*	*
Hamza et al., 2006 [31] Tanzania	Cross-sectional	481 *	*	276	1–14 months	< 200 cells/mm^3^ (information regarding 338 patients receiving HAART)	*
Jané-Salas et al., 2006 [21] Spain	Cross-sectional	90 (51 ♂ 39 ♀)	Injectable drugs (47) Sexual intercourse (27)	90	*	*	*
Cepeda et al., 2008 [44] Spain	Cross-sectional	86 (42 ♂ 44 ♀)	Sexual intercourse (53) Injectable drugs (33)	86	*	*	Undetectable (39) < 10,000 copies/mL (25) > 10,000 copies/mL (22)
Lourenço and Figueiredo, 2008 [33] Brazil	Cross-sectional	340 (217 ♂ 123 ♀)		221		*	*
Ortega et al., 2008 [38] Spain	Cross-sectional	105 (75 ♂ 30 ♀)	Sexual intercourse (49) Injectable drugs (46)	105	*	*	*
Nittayananta et al., 2010 [37] Thailand	Cross-sectional	157 (71 ♂ 86 ♀)	Sexual intercourse (141)	99	< 3 years (45) ≥ 3 years (54)	5–699 cells/mm^3^ (58 without HAART) 9–630 cells/mm^3^ (45 in < 3 years of HAART) 74–1,600 cells/mm^3^ (54 in ≥ 3 years of HAART)	0–30,000 copies/mL (58 without HAART) 50–750,000 copies/mL (45 in < 3 years of HAART) 50–139,000 (54 in ≥ 3 years of HAART)
Taiwo and Hassan, 2010 [40] Nigeria	Cross-sectional	142 (42 ♂ 80 ♀)	*	142	*	Before HAART = 148.5 ± 117.5 cells/mL After 24 weeks of HAART = 253.87 ± 147 cells/mL	Before HAART = 163,831.9 ± 279,964.9 copies/mL After 24 weeks of HAART = 13,900.28 ± 52,352 copies/mL
Casariego et al., 2012 [22] Argentina	Cross-sectional	2611 *	*	2611	*	*	*
Eweka et al., 2012 [42] Estados Unidos	Cross-sectional	114 (49 ♂ 65 ♀)	*	114	3 months	*	*
Freeman et al., 2012 [30] Australia	Cross-sectional	495 (471 ♂ 24 ♀)	*	367	*	*	*
Perera et al., 2012 [43] China	Cross-sectional	101 (87 ♂ 14 ♀)	Sexual intercourse (95), blood transfusion, and intravenous drug use (6)	101	> 24 meses	< 200 cells/mL (23) 200–500 cells/mL (52) > 500 cells/mL (26)	< 50 copies/ml (59) 50–10,000 copies/ml (33) > 10,000 copies/ml (9)
Ricardo et al., 2012 [23] Colombia	Cross-sectional	72	*	72	*	*	*
Mthethwa et al., 2013 [34] South Africa	Cross-sectional	203 (64 ♂ 137 ♀) Information not available for 2 patients	*	140	1–3 months (50) 4–14 months (103) 15–25 months (50)	Patients with HAART < 100 cells/mm^3^ (38) 100–200 cells/mm^3^ (27) > 200 cells/mm^3^ (63) Patients without HAART < 100 cells/mm^3^ (22) 100–200 cells/mm^3^ (25) > 200 cells/mm^3^ (6)	*
Naidu et al., 2013 [35] Nepal	Cross-sectional	81 (54 ♂ 27 ♀)	Injectable drugs (31) Sexual intercourse (25)	28	*	Patients with HAART 190 cells/mm^3^ (28) Patients without HAART 350.3 cells/mm^3^ (53)	*
Patil et al., 2015 [39] India	Cross-sectional	100 (56 ♂ 44 ♀)	*	50	*	Patients without HAART 258.82 cells/mm^3^ (50) Patients with HAART 303.68 cells/mm^3^ (50)	*
Rao et al., 2015 [45] India	Cross-sectional	320 (178 ♂ 142 ♀)	Sexual intercourse (233) Unknown causes (45) Blood transfusion (30) Vertical transmission (12)	320	3 months	After 3 months of HAART 150–200 cells/mm^3^ (57) 200–250 cells/mm^3^ (186) 250–300 cells/mm^3^ (77)	*
Arirachakaran et al., 2016 [25] Thailand	Cross-sectional	168 (64 ♂ 104 ♀)	*	148	*	Patients with HAART 439 +—207 cells/mm^3^ Patients without HAART 452 +—202 cells/mm^3^	Patients with HAART < 50 copies/ml Patients without HAART 2,000–350,000 copies/ml
Denny et al., 2016 [27] India	Cross-sectional	108 (64 ♂ 44 ♀)	*	108	*	*	*
Dongade et al., 2018 [28] India	Cross-sectional	373 (206 ♂ 167 ♀)	*	373	*	*	*
Abe et al., 2021 [24] Nigeria	Cross-sectional	227 (173 ♂ 54 ♀)	Sexual intercourse (178) Blood transfusion (5) Mother to child (5) Unknown reasons (40)	227	2–5 years (23) 6–10 years (40) > 10 years (38)	< 200 cells/mm^3^ (59) 200–500 cells/mm^3^ (109) > 500 cells/mm^3^ (59)	*
Bartholo et al., 2023 [26] Brazil	Cross-sectional	101 (62 ♂ 74 ♀)	Sexual intercourse (84) Injectable drugs (5) Blood transfusion (2) Multiple risk factors (3) Vertical (2) Unknown (2)	101	< 10 years (27) 10–20 years (46) > 20 years (28)	< 200 cells/mm^3^ (8) 200–499 cells/mm^3^ (18) ≥ 500 cells/mm^3^ (71)	Undetectable (94) ≥ 10,000 copies/mL (3)

^*^Information not available

The articles presented heterogeneity in the sample’s methodological design and the analysis of oral lesion prevalence. The samples of 11 articles consisted of groups of HIV-positive patients receiving and not receiving HAART. In 11 articles, the sample consisted of only one group of HIV-positive patients under HAART. Three articles analyzed oral lesion prevalence in a group of HIV-positive patients before and after receiving HAART. The analysis of oral manifestations showed a higher prevalence of pseudomembranous and erythematous candidiasis, angular cheilitis, oral hairy leukoplakia, oral mucosa hyperpigmentation, and recurrent oral ulceration. Table 3 details the prevalence of oral lesions in HIV-positive patients receiving and not receiving HAART.

**Table 3:** Prevalence of oral manifestations in HIV-positive patients

Studies presenting the prevalence of oral manifestations in HIV-positive patients receiving and not receiving HAART														
**Author and year**	**Sample**	**Oral manifestations**												
		**Pseudomembranous candidiasis**	**Erythematous candidiasis**	**Angular cheilitis**	**Herpes simplex**	**Herpes zoster**	**Kaposi's sarcoma**	**Oral hairy leukoplasia**	**Oral mucosa hyperpigmentation**	**Oral warts**	**Recurrent oral ulceration**	**Salivary gland diseases**	**Necrotizing ulcerative gingivitis**	**Bell's palsy**
Tappuni and Fleming, 2001 [41]	231 NH: 195 WH: 36	NH: 21 WH: 3	NH: 41 WH: 3	*	*	*	*	NH: 16 WH: 2	*	*	*	*	NH: 12 WH: 0	*
Eyeson et al., 2002 [29]	192 NH: 51 WH: 141	NH: 3 WH: 4	NH: 3 WH: 4	NH: 3 WH: 11	*	*	NH: 0 WH: 4	NH: 9 WH: 8	*	*	NH: 11 WH: 34	*	NH: 4 WH; 4	*
Nicolatou-Galitis et al., 2004 [36]	81 NH: 37 WH: 44	NH: 9 WH: 5	NH: 4 WH: 3	*	NH: 1 WH: 1	NH: 0 WH: 1	*	NH: 3 WH: 4	*	*	*	NH: 2 WH: 3	NH: 3 WH: 0	*
Hamza et al., 2006 [31]	481 NH: 205 WH: 276	*	*	*	NH: 5 WH: 3	NH: 2 WH: 0	NH: 10 WH: 5	NH: 8 WH: 2	NH: 8 WH: 17	NH: 1 WH: 2	NH: 3 WH: 1	NH: 5 WH: 6	NH: 8 WH: 5	NH: 3 WH: 0
Lourenço and Figueiredo, 2008 [33]	340 NH: 69 WH: 271	NH: 14 WH: 17	NH: 7 WH: 13	NH: 12 WH: 28	NH: 2 WH: 1	*	NH: 1 WH: 0	NH: 18 WH: 22	NH: 0 WH: 1	*	NH: 1 WH: 6	*	*	*
Nittayananta et al., 2010 [37]	157 NH: 58 WH: 99	NH: 1 WH: 2	NH: 4 WH: 0	*	*	*	*	NH: 3 WH: 1	NH: 16 WH: 44	*	NH: 2 WH: 3	*	NH: 2 WH: 1	*
Freeman et al., 2012 [30]	495 NH: 128 WH: 367	*	*	NH: 5 WH: 15	*	*	NH: 3 WH: 4	NH: 29 WH: 55	*	*	NH: 5 WH: 21	NH: 1 WH: 6	*	*
Mthethwa et al., 2013 [34]	203 NH: 63 WH: 140	NH: 15 WH: 10	NH: 5 WH: 10	*	*	*	*	NH: 1 WH: 0	*	*	*	NH: 2 WH: 2	NH: 6 WH: 6	*
Naidu et al., 2013 [35]	81 NH: 53 WH: 28	NH: 1 WH: 3	NH: 9 WH: 4	NH: 1 WH: 0	NH: 6 WH: 0	*	NH: 0 WH: 0	NH: 6 WH: 4	NH: 8 WH: 9	NH: 0 WH: 0	NH: 1 WH: 0	*	NH: 4 WH: 3	NH: 2 WH: 1
Patil et al., 2015 [39]	100 NH: 50 WH: 50	NH: 8 WH: 2	NH: 4 WH: 0	NH: 4 WH: 0	*	*	*	*	NH: 10 WH: 14	*	NH: 9 WH: 8	*	*	*
Arirachakaran et al., 2016 [25]	168 NH: 20 WH: 148	NH: 0 WH: 2	*	NH: 0 WH: 4	NH: 0 WH: 2	*	*	NH: 1 WH: 2	*	*	NH: 4 WH: 17	*	*	*

**Studies presenting the prevalence of oral manifestations only in HIV-positive patients receiving HAART**														
**Author and year**	**Sample**	**Oral manifestations**												
		**Pseudomembranous candidiasis**	**Erythematous candidiasis**	**Angular cheilitis**	**Herpes simplex**	**Herpes zoster**	**Kaposi's sarcoma**	**Oral hairy leukoplasia**	**Oral mucosa hyperpigmentation**	**Oral warts**	**Recurrent oral ulceration**	**Salivary gland diseases**	**Necrotizing ulcerative gingivitis**	**Bell's palsy**
Kroidl et al., 2005 [32]	129	*	*	*	4	*	1	5	*	*	2	*	2	*
Jané-Salas et al., 2006 [21]	90	*	28	2	4	*	3	1	*	*	*	*	*	*
Ortega et al., 2008 [38]	105	*	*	3	1	*	*	1	*	*	1	12	*	*
Taiwo and Hassan, 2010 [40]	142	4	9	3	1	*	3	6	12	*	4	3	*	*
Casariego et al., 2012 [22]	2611	517	603	170	85	*	89	251	*	*	502	*	108	*
Perera et al., 2012 [43]	101	*	*	1	*	*	2	2	3	*	3	2	*	*
Ricardo et al., 2012 [23]	82	47	6	*	*	*	*	*	*	*	2	*	*	*
Denny et al., 2016 [27]	108	*	*	*	3	*	0	4	46	*	6	*	*	*
Dongade et al., 2018 [28]	373	*	*	*	*	4	*	4	166	*	6	*	*	*
Abe et al., 2021 [24]	227	*	*	*	*	*	*	*	43	*	2	*	*	*
Bartholo et al., 2023 [26]	101	2	3	4	*	*	1	1	4	*	1	12	*	*

**Studies presenting the prevalence of oral manifestations in HIV-positive patients before and after HAART**														
**Author and year**	**Sample**	**Oral manifestations**												
		**Pseudomembranous candidiasis**	**Erythematous candidiasis**	**Angular cheilitis**	**Herpes simplex**	**Herpes zoster**	**Kaposi's sarcoma**	**Oral hairy leukoplasia**	**Oral mucosa hyperpigmentation**	**Oral warts**	**Recurrent oral ulceration**	**Salivary gland diseases**	**Necrotizing ulcerative gingivitis**	**Bell's palsy**
Cepeda et al., 2008 [44]	86 Pre: 86 Post: 86	Pre: 11 Post: 4	Pre: 27 Post: 25	Pre: 20 Post: 8	Pre: 7 Post: 1	*	Pre: 4 Post: 0	Pre: 18 Post: 20	*	*	*	*	*	*
Eweka et al., 2012 [42]	114 Pre: 114 Post: 114	*	*	*	*	*	*	Pre: 14 Post: 0	Pre: 7 Post: 2	*	*	*	*	*
Rao et al., 2015 [45]	320 Pre: 320 Post: 320	Pre: 12 Post: 4	Pre: 36 Post: 19	Pre: 22 Post: 9	Pre: 1 Post: 0	*	*	*	Pre: 17 Post: 76	*	Pre: 4 Post: 3	*	*	*

*NH* No HAART, *WH* With HAART, *Pre* Pre-HAART, *Post* Post-HAART

^*^Variables not analyzed or information not obtained

### Risk of bias in studies

**Fig. 2:**
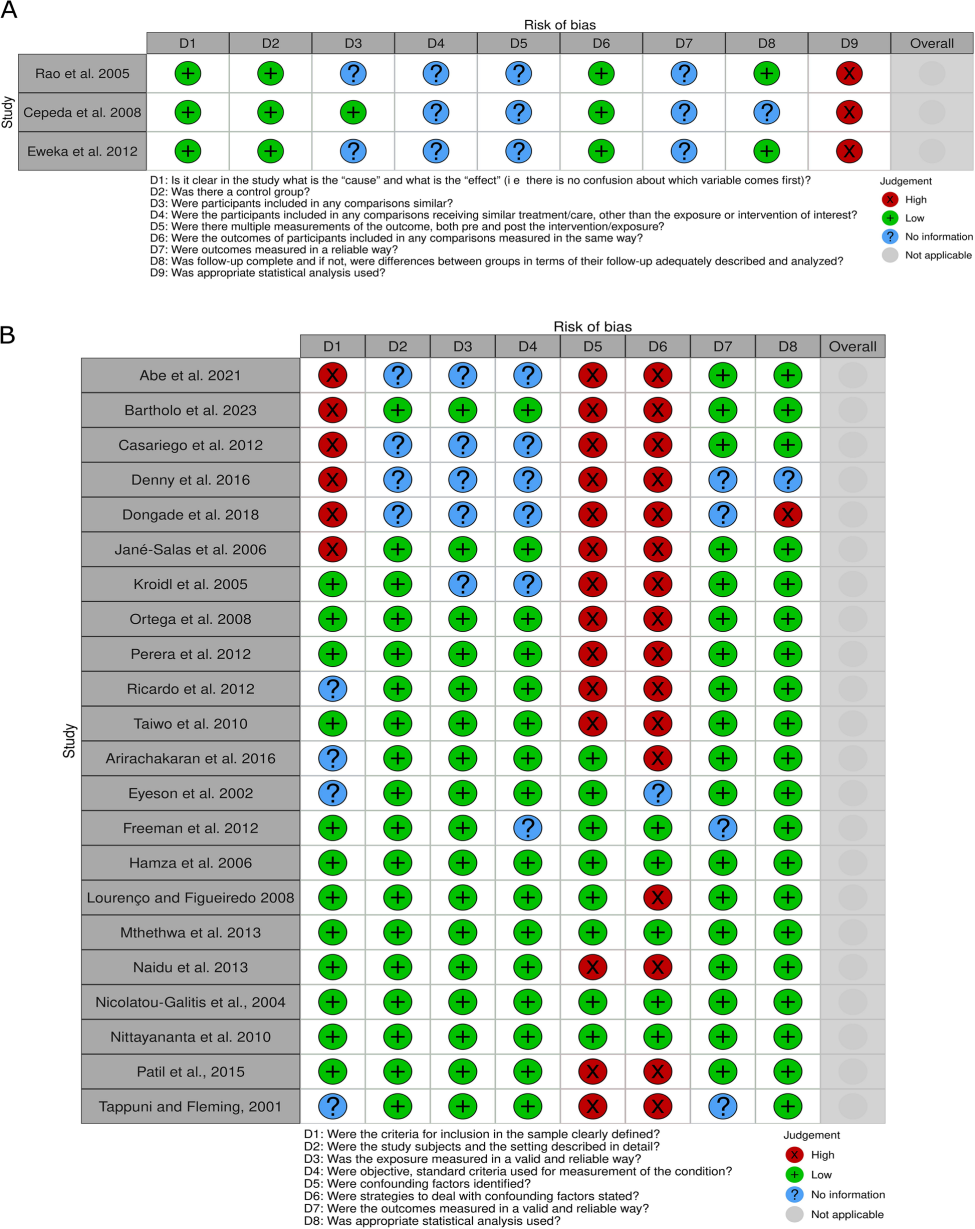
Risk of bias assessment. **a** Before-and-after studies **b** Cross-sectional studies

The three studies comparing patient outcomes before and after initiation of HAART exhibited significant sources of bias, stemming from insufficient clarity regarding exposure to treatments other than HAART, the presence of multiple comparisons, and the lack of an adequate description of outcome assessment methods [44, 45]. Additionally, all studies conducted inadequate statistical analyses for paired samples (Fig. 2a). In the cross-sectional studies, the primary sources of bias were the failure to identify confounding factors and the insufficient clarity in reporting sample eligibility criteria. Only four studies presented a low risk of bias in all evaluated domains (Fig. 2b).

### Synthesis of results and certainty of evidence

**Fig. 3:**
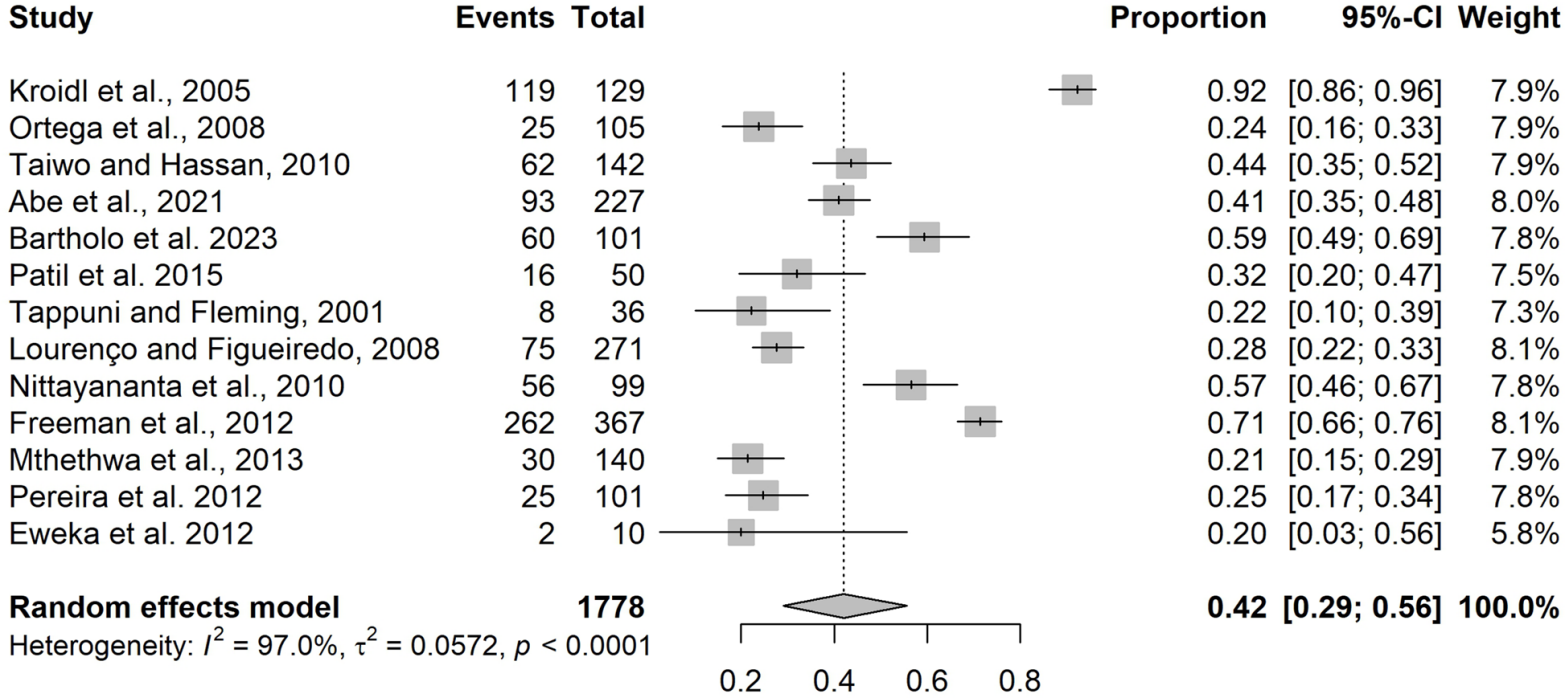
Meta-analysis of proportions demonstrating the prevalence of oral manifestations in patients receiving HAART

**Fig. 4:**
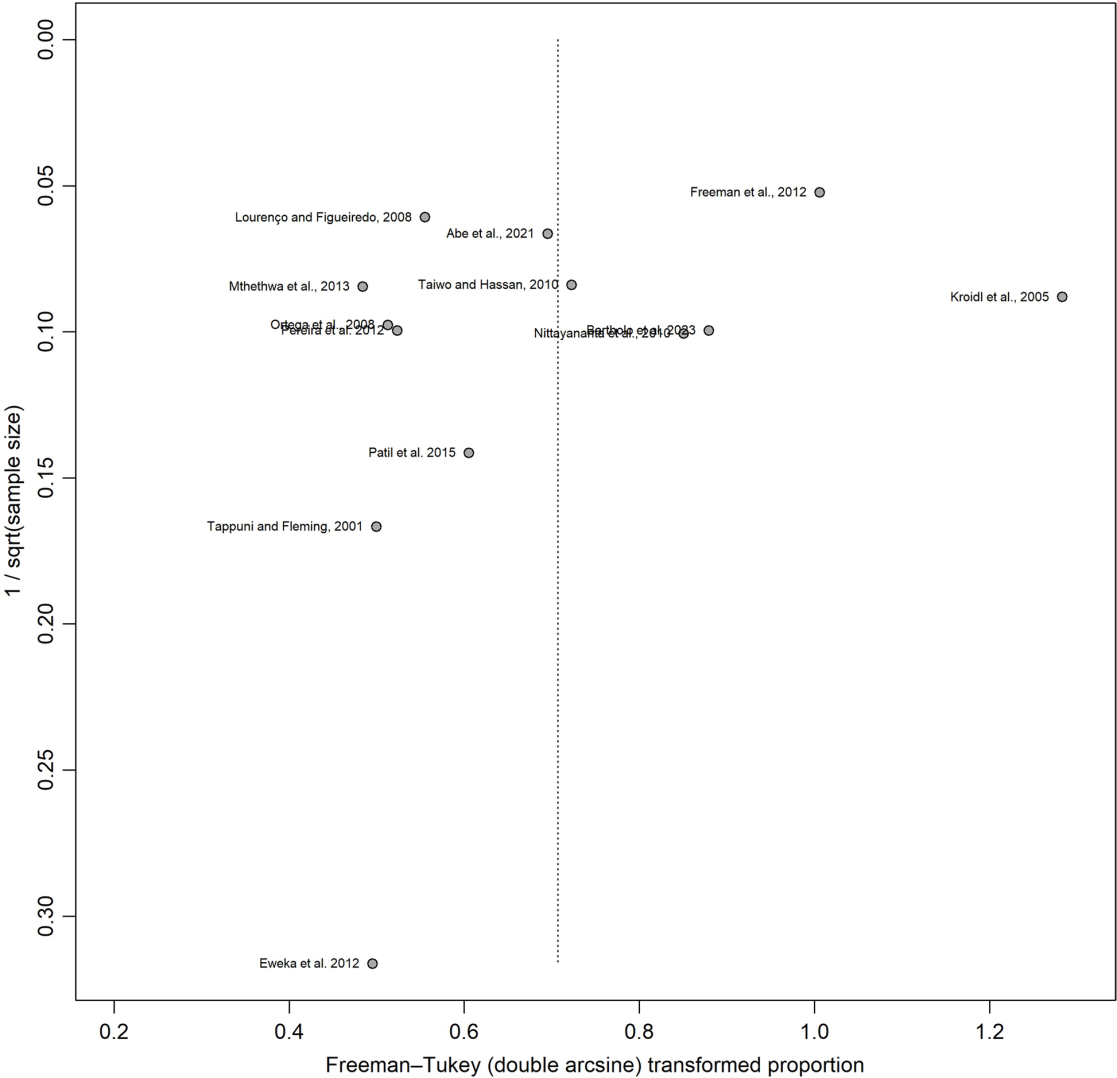
Funnel plot

The analysis of oral lesion prevalence in patients receiving HAART included 13 studies. This analysis revealed a prevalence of 42% (95% CI: 29% – 56%, *n* = 1778), with high heterogeneity (I^2^ = 97%, *p* < 0.001, tau^2^ = 0.0572) (Fig. 3). The funnel plot (Fig. 4) showed no asymmetry, as indicated by the Egger test (*p* = 0.37). Oral mucosa hyperpigmentation was the most common finding (16.2%; 95% CI: 7.7–27.0), while herpes simplex was least common (1.2%; 95% CI: 0.4–2.2) (Supplementary Table 2).

**Fig. 5:**
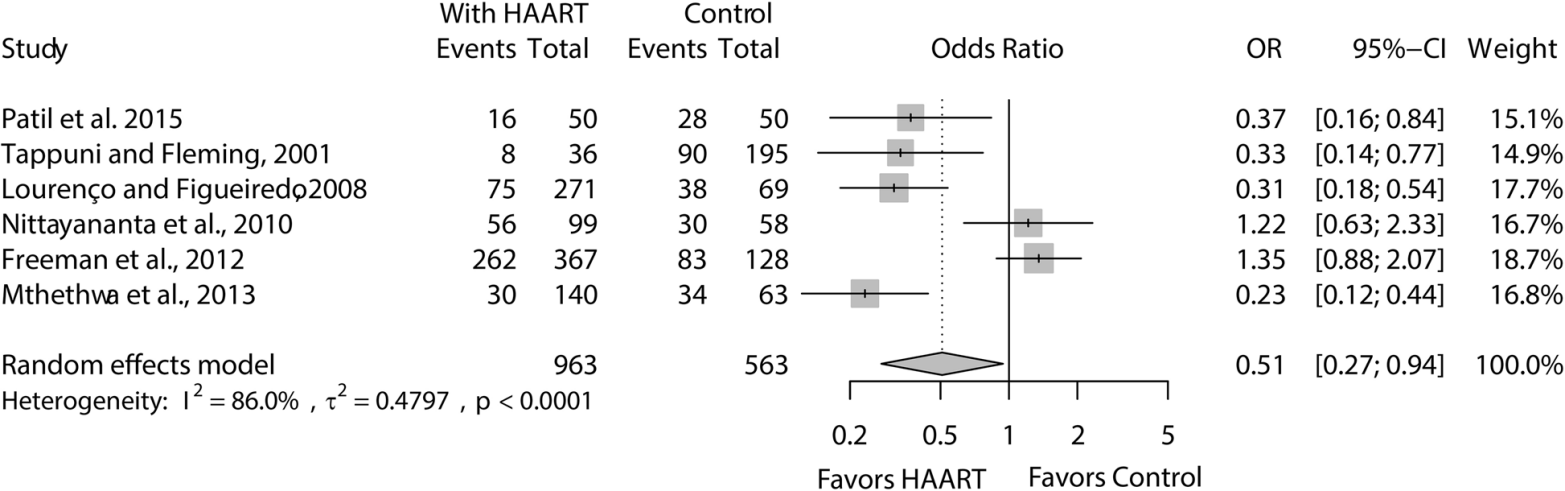
Meta-analysis of comparisons of the chances of patients receiving and not receiving HAART to present oral

Regarding the direct comparisons among patients receiving and not receiving HAART, the meta-analysis based on six studies showed that patients using the drug were less likely to present oral manifestations (OR: 0.51; 95% CI: 0.27–0.94, *p* = 0.03) (Fig. 5). This result exhibited high heterogeneity (I^2^ = 86%, *p* < 0.001, tau^2^ = 0.47) and very low certainty of evidence (Table 4). After excluding high-risk bias studies, this comparison was no longer significant (OR: 0.74; 95% CI: 0.24–2.24, *p* = 0.59) and showed substantial heterogeneity (I^2^ = 90.7%, *p* < 0.001, tau^2^ = 0.88) (Supplementary Fig. 1).

**Table 4:** Summary of findings table

Outcome	Patients (studies), N	Relative effect (95% CI)	**Absolute effects (95% CI)**			Certainty	Summary
			**Without treatment**	**HAART**	**Diferença**		
Oral lesions	1526 (6 observational studies)	OR = 0.51 (0.27 to 0.94)	538 by 1.000	373 by 1.000 (239 to 523)	165 less by 1.000 (from 299 to 15 less)	⨁◯◯◯ Very Low^a^	HAART may reduce/have moderate effect on oral lesions prevalence, but the evidence is very uncertain

*CI* Confidence interval, *OR* Odds ratio

^a^Evidence of serious inconsistency (I.^2^ > 75%)

## Discussion

### Sumary of main results

This systematic review aimed to evaluate the influence of highly active antiretroviral therapy (HAART) on the prevalence of oral lesions in adult patients with human immunodeficiency virus (HIV). The meta-analysis showed that patients receiving HAART were less likely to present oral manifestations than HIV-positive patients not receiving this therapy. However, due to the high degree of heterogeneity of the studies, the findings should be interpreted with caution.

HIV is a single-stranded RNA virus that progressively damages the host's immune system by destroying CD4 + T cells and preventing the body from fighting opportunistic infections [46]. Shaw and Hunter (2012) highlight unprotected sexual intercourse and injectable drugs as the main transmission routes of this virus, which agrees with the HIV transmission routes mentioned in the articles included in this review [47].

The introduction of antiretroviral therapies, such as HAART, significantly reduced the viral load, partially restored the immune system, and helped decrease mortality and increase life expectancy among HIV-positive individuals [48]. HAART is a treatment method that combines three or more antiretroviral drugs, including two nucleoside reverse transcriptase inhibitors plus one non-nucleoside reverse transcriptase inhibitor, one integrase inhibitor, or one protease inhibitor that was enhanced by ritonavir or cobicistat [49, 50]. One of the main objectives in co-administering these drugs is to inhibit the spread of a virus resistant to a single agent through the action of the other two agents [51].

Oral manifestations are early indicators of HIV infection and may predict the progression of this infection [52]. Lomelí-Martínez (2022) found that oral manifestations with the highest prevalence in HIV-positive patients are due to fungal infections such as oral candidiasis, herpes simplex viral infections, oral mucosal hyperpigmentation, periodontal disease, oral hairy leukoplakia, and Kaposi's sarcoma, which agrees with the oral manifestations described in the articles included in this review [53]. The introduction of HAART significantly reduced these oral lesions [54]. The direct comparison meta-analysis performed in the present study showed that HIV-positive patients receiving HAART were less likely to present any oral manifestation. However, it is worth noting that, despite the lower manifestation of these conditions, the prevalence analysis found oral lesions in 42% of patients receiving HAART.

Despite the reduction in oral lesion prevalence, the impact of HAART was not uniform across all included studies. In five studies, candidiasis remained more prevalent among patients receiving HAART [25, 29, 30, 33, 37]. Patton (2014) reports that candidiasis accounts for the highest prevalence of lesions in HIV-positive patients, regardless of HAART application [55]. The prolonged application of this therapy may cause side effects and be associated with xerostomia and salivary changes that may contribute to the persistence or re-emergence of this lesion [24].

Oral mucosal hyperpigmentation was also more prevalent in HIV-positive patients under HAART [31, 33, 35, 37, 39]. Hamza et al. (2006) affirm that the increased oral mucosa hyperpigmentation in these patients occurs as a consequence of zidovudine, which is present in HAART, by stimulating the release of the melanocyte-stimulating hormone and increasing melanin production in the oral epithelium [31]. However, Vasudevan et al. (2012) report that melanocyte stimulation occurs through immunopathological changes [56].

The results of some included studies showed that HAART reduced the manifestation of Kaposi's sarcoma [21, 22, 31–33, 40, 44]. This condition consists of a vascular endothelial neoplasm first described in 1972 by Moritz Kaposi [57], caused by the human herpesvirus type 8 (HHV-8), and representing one of the most common neoplasms among HIV-positive individuals who have developed the acquired immunodeficiency syndrome [58]. In HIV-positive patients also infected with HHV-8, the HIV transactivating protein and the HIV-1 negative factor interact with the HHV-8 viral interleukin, stimulating angiogenesis and tumorigenesis, which explains the aggressive course of this tumor in AIDS patients [59]. Introducing HAART in HIV management enhances immunological defense mechanisms and the host response to HHV-8. Consequently, it reduces the incidence of this condition, facilitates its regression or complete remission, decreases mortality, and explains the lower prevalence of Kaposi's sarcoma reported in the studies included in this systematic review [60–62].

The manifestation of oral hairy leukoplakia also decreased in patients receiving HAART [26]. Clinically, it presents as non-removable whitish plaques often located on the lateral edges of the tongue [63]. In HIV-positive patients, this lesion is associated with the coinfection by the Epstein-Barr virus, occurring with higher intensity in immunosuppressed patients, and it was highly prevalent before the introduction of antiretroviral therapies [64]. Although HAART provides conditions for immune function recovery, HIV-positive patients receiving this therapy had lower CD4 + T lymphocyte counts than those not receiving it. Naidu et al. (2013) also found an increase in the prevalence of this lesion. These authors believe this condition occurs due to the protocol of introducing HAART only when patients had a CD4 cell count below 200 cells/mm [3], which directly influences immunosuppression and promotes higher vulnerability to coinfection by the Epstein-Barr virus [35].

Patients using HAART did not experience a reduction in the manifestations of recurrent oral ulceration [25, 29, 30, 33, 37]. This lesion may occur due to prolonged stress with consequent influence on the hypothalamic-pituitary–gonadal axis [65]. HIV-positive patients are often under pharmacological and emotional stress, which may explain the frequent presence of these ulcerations in the oral mucosa [65].

Although HAART influences the prevalence of periodontal diseases, some studies included in this review did not find a significant reduction in this condition [31, 35–37, 41]. The progression of these diseases may be related to local hygiene factors and the status of the immune system [66]. CD4 + T cell count is one of the most evident indicators of immune system impairment [54]. Mutoh et al. (2018) [67] found that levels lower than 200 CD4 +/mm^3^ T cells may be related to the progression of opportunistic infections, which was visible in HIV-positive patients receiving HAART, potentially explaining the non-significant reduction of periodontal disease in these studies [31, 35–37, 41].

Salivary gland diseases were not highly prevalent in the studies included in this review, regardless of the application of HAART. Parotid gland enlargement may be associated with CD8 lymphocyte infiltration, known as diffuse infiltrative lymphocytic syndrome, and it occurs in HIV-positive patients with low CD4 + T-cell counts and an impaired immune system [68].

Bell's palsy appeared in only two studies [31, 35]. It is an acute idiopathic paralysis of the facial nerve and represents approximately half of all cases of peripheral paralysis of this nerve. HIV-positive patients can manifest acute retroviral syndrome and trigger episodes of fever, myalgia, headaches, skin rashes, and lymphadenopathy soon after primary exposure to HIV, at the onset of the seroconversion process [69]. Kandah et al. (2023) report that most associations with facial nerve palsy and HIV infection are seen at the time of seroconversion [70]. However, these authors presented a case in which the patient manifested Bell's palsy late and with a CD4 + T cell count of 172 cells/mm^3^, which also agrees with the CD4 + T cell count below 200 cells/mm^3^ in the patients of the studies by Hamza et al. (2006) [31] and Naidu et al. (2013) [35].

### Potential biases in the review process

Although the present review demonstrated that HAART provided a lower chance of oral lesions in HIV-positive patients, the findings should be interpreted with caution. The included studies present a high degree of heterogeneity regarding the methodological design, the presence of patients with diverse viral loads and CD4 + cell counts in the same sample, the time of HAART application, and the types of active ingredients in this therapy.

### Agreements and disagreements with other reviews

The results of the systematic review conducted by Almeida et al. (2017) showed that oral manifestations of candidiasis, herpes simplex infection, and oral lesions such as Kaposi’s sarcoma and oral hairy leukoplakia had a significantly lower prevalence in HIV-positive patients undergoing HAART [71]. Although these findings are consistent with the results of the present study, it is worth noting that a large proportion of the studies included in both reviews presented a moderate to high risk of bias due to failures in identifying confounding factors and insufficient clarity in describing the sample eligibility criteria. Moreover, the very low certainty of evidence requires caution in the interpretation of results and underlines the urgency for primary studies with standardized methodologies.

## Conclusion

HAART contributes to the reduction of oral manifestations in HIV-positive patients; however, it is essential that future primary studies adopt standardized methodologies to ensure greater reliability and accuracy of the evidence obtained. It is critical to empower healthcare professionals to recognize the direct effects of HIV infection and the oral consequences associated with antiretroviral treatment, ensuring integrated and patient-centered care.

## Supplementary Information

Supplementary Material 1.

Supplementary Material 2.

Supplementary Material 3.

## Data Availability

All data generated or analysed during this study are included in this published article.

## Abbreviations

HAART: Highly active antiretroviral therapy
HIV: Human immunodeficiency virus
PRISMA: Preferred Reporting Items for Systematic Reviews and Meta-Analyses
PROSPERO: Prospective International Registry of Systematic Reviews
JBI: Joanna Briggs Institute Manual
Cis: Confidence intervals
Ors: Odds Ratios
GRADE: Grading of Recommendations, Assessment, Development, and Evaluation
