# Supplementary material for: Oral manifestations in HIV-positive individuals under highly active antiretroviral therapy: a systematic review and meta-analysis of prevalence data
Source: BMC Oral Health. 2026 Mar 31;26:816. doi: 10.1186/s12903-026-08182-0 (PMC13159240; doi:10.1186/s12903-026-08182-0)
Supplement: Supplementary file 1 — Supplementary Material 1. [file 12903_2026_8182_MOESM1_ESM.pdf]

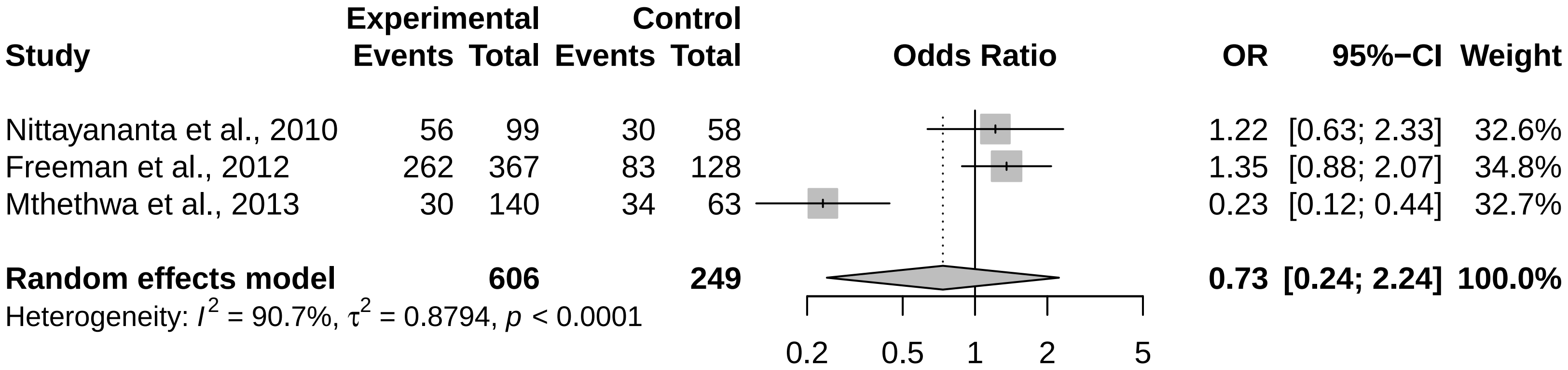

Sensitivity analysis of comparisons of the chances of patients receiving and not receiving HAART to present oral.
