# Supplementary material for: Oral manifestations in HIV-positive individuals under highly active antiretroviral therapy: a systematic review and meta-analysis of prevalence data
Source: BMC Oral Health. 2026 Mar 31;26:816. doi: 10.1186/s12903-026-08182-0 (PMC13159240; doi:10.1186/s12903-026-08182-0)
Supplement: Supplementary file 2 — Supplementary Material 2. [file 12903_2026_8182_MOESM2_ESM.docx]

**Supplementary Table 1** – Reasons for exclusions

| Ceballos-Salobreña et al., 2000^1^ | HIV-positive patients on antifungal therapy |
| --- | --- |
| Birbaum et al., 2002^2^ | Review article |
| King et al., 2002^3^ | The patients presented with HIV and HPV infection |
| Greenspan et al., 2004^4^ | HIV-positive patients on antifungal therapy |
| Pinheiro et al., 2004^5^ | Artigo de Revisão |
| Cameron et al., 2005^6^ | The patients presented with HIV and HPV infection |
| Hodgson et al., 2006^7^ | Review article |
| Silva et al., 2008^8^ | There is no possibility of distinguishing the measurement of variables between adults and children |
| Cavasin Filho and Giovani, 2009^9^ | The variable analyzed in the study is not consistent with the variables analyzed in the present review |
| Gaitán-Cepeda et al., 2010^10^ | Sample consisting of children and adolescents |
| Nittayananta et al., 2010^11^ | The variable analyzed in the study is not consistent with the variables analyzed in the present review |
| Jimenes et al., 2012^12^ | HIV-positive patients receiving antiretroviral therapy that does not qualify as HAART |
| Anaya-Saavedra et al., 2013^13^ | The patients presented with HIV and HPV infection |
| Camacho-Aguilar et al., 2018^14^ | The patients presented with HIV and HPV infection |
| El-Mallawahy et al., 2016^15^ | Sample consisting of children and adolescents |
| Yengopal et al., 2016^16^ | Sample consisting of children and adolescents |
| Arubaku et al., 2022^17^ | The variable analyzed in the study is not consistent with the variables analyzed in the present review |
| Nittayananta et al., 2016^18^ | Review article |
| Ricardo et al., 2016^19^ | HIV-positive patients receiving antiretroviral therapy that does not qualify as HAART |
| Shintani et al., 2019^20^ | The variable analyzed in the study is not consistent with the variables analyzed in the present review |

**References**

1. [Ceballos-Salobreña](https://pubmed.ncbi.nlm.nih.gov/?term=Ceballos-Salobre%C3%B1a+A&cauthor_id=11119429), A., [Gaitán-Cepeda](https://pubmed.ncbi.nlm.nih.gov/?term=Gait%C3%A1n-Cepeda+LA&cauthor_id=11119429), L. A., [Ceballos-Garcia](https://pubmed.ncbi.nlm.nih.gov/?term=Ceballos-Garcia+L&cauthor_id=11119429), L.,  [Lezama-Del Valle](https://pubmed.ncbi.nlm.nih.gov/?term=Lezama-Del+Valle+D&cauthor_id=11119429), D. Oral lesions in HIV/AIDS patients undergoing highly active antiretroviral treatment including protease inhibitors: a new face of oral AIDS? AIDS Patient Care STDS **14**(12), 627-635. https://doi.org/doi: 10.1089/10872910050206540 (2000).
2. Birnbaum W, Hodgson TA, Reichart PA, Sherson W, Nittayananta, W., Axell, T. E. Prognostic significance of HIV-associated oral lesions and their relation to therapy. Oral Dis. **8** Suppl 2, 110-114. <https://doi.org/10.1034/j.1601-0825.2002.00021.x> (2002).
3. [King](https://pubmed.ncbi.nlm.nih.gov/?term=King+MD&cauthor_id=11803508), M. D., [Reznik](https://pubmed.ncbi.nlm.nih.gov/?term=Reznik+DA&cauthor_id=11803508), D. A., [O'Daniels](https://pubmed.ncbi.nlm.nih.gov/?term=O%27Daniels+CM&cauthor_id=11803508), C. M., [Larsen](https://pubmed.ncbi.nlm.nih.gov/?term=Larsen+NM&cauthor_id=11803508), N. M., Osterholt, D., Blumberg, H. M. Human papillomavirus-associated oral warts among human immunodeficiency virus-seropositive patients in the era of highly active antiretroviral therapy: an emerging infection. Clin. Infect. Dis. **34**(5), 641-648. <https://doi.org/10.1086/338637> (2002).
4. [Greenspan](https://pubmed.ncbi.nlm.nih.gov/?term=Greenspan+D&cauthor_id=14742653), D., et al. Incidence of oral lesions in HIV-1-infected women: reduction with HAART. J. Dent. Res. **83**(2), 145 150. <https://doi.org/10.1177/154405910408300212> (2004).
5. [Pinheiro](https://pubmed.ncbi.nlm.nih.gov/?term=Pinheiro+A&cauthor_id=15218892), [A](https://pubmed.ncbi.nlm.nih.gov/15218892/#full-view-affiliation-1)., [Marcenes](https://pubmed.ncbi.nlm.nih.gov/?term=Marcenes+W&cauthor_id=15218892), W., [Zakrzewska](https://pubmed.ncbi.nlm.nih.gov/?term=Zakrzewska+JM&cauthor_id=15218892), J. M., [Robinson](https://pubmed.ncbi.nlm.nih.gov/?term=Robinson+PG&cauthor_id=15218892), P. G. Dental and oral lesions in HIV infected patients: a study in Brazil. Int. Dent. J. **54**(3), 131-137. <https://doi.org/10.1111/j.1875-595x.2004.tb00268.x> (2004).
6. [Cameron](https://pubmed.ncbi.nlm.nih.gov/?term=Cameron+JE&cauthor_id=16254546), J. E., et al. The impact of highly active antiretroviral therapy and immunodeficiency on human papillomavirus infection of the oral cavity of human immunodeficiency virus-seropositive adults. Sex. Transm. Dis. **32**(11), 703-709. <https://doi.org/10.1097/01.olq.0000175398.34610.2e> (2005).
7. [Hodgson](https://pubmed.ncbi.nlm.nih.gov/?term=Hodgson+TA&cauthor_id=16672551), T. A., [Greenspan](https://pubmed.ncbi.nlm.nih.gov/?term=Greenspan+D&cauthor_id=16672551), D., [Greenspan](https://pubmed.ncbi.nlm.nih.gov/?term=Greenspan+JS&cauthor_id=16672551), J. S. Oral lesions of HIV disease and HAART in industrialized countries. Adv. Dent. Res. **19**(1), 57-62. <https://doi.org/10.1177/154407370601900112> (2006).
8. [Silva](https://pubmed.ncbi.nlm.nih.gov/?term=da+Silva+CA&cauthor_id=18248345), C. A. L., [Dourado](https://pubmed.ncbi.nlm.nih.gov/?term=Dourado+I&cauthor_id=18248345), I., [Dahia](https://pubmed.ncbi.nlm.nih.gov/?term=Dahia+SR&cauthor_id=18248345), S. R., [Harzheim](https://pubmed.ncbi.nlm.nih.gov/?term=Harzheim+E&cauthor_id=18248345), E. Oral manifestations of HIV infection in patients receiving highly active antiretroviral therapy (HAART) in Bahia, Brazil. J. Public Health Dent. **68**(3), 178-181. <https://doi.ORG/10.1111/j.1752-7325.2007.00071.x> (2008).
9. Cavasin [Filho](https://pubmed.ncbi.nlm.nih.gov/?term=Cavasin+Filho+JC&cauthor_id=19578624), J. C. C., [Giovani](https://pubmed.ncbi.nlm.nih.gov/?term=Giovani+EM&cauthor_id=19578624), E. M. Xerostomy, dental caries and periodontal disease in HIV+ patients. Braz J Infect Dis 13:13-7. <https://doi.org/10.1590/s1413-86702009000100005> (2009).
10. [Gaitán-Cepeda](https://pubmed.ncbi.nlm.nih.gov/?term=Gait%C3%A1n-Cepeda+LA&cauthor_id=20173726), L. A., et al. Oral lesions in HIV+/AIDS adolescents perinatally infected undergoing HAART. Med Oral Patol Oral Cir Bucal 15(4), e545-550. (2010).
11. [Nittayananta](https://pubmed.ncbi.nlm.nih.gov/?term=Nittayananta+W&cauthor_id=19709350), W., [Chanowanna](https://pubmed.ncbi.nlm.nih.gov/?term=Chanowanna+N&cauthor_id=19709350), N., [Jealae](https://pubmed.ncbi.nlm.nih.gov/?term=Jealae+S&cauthor_id=19709350), S., [Nauntofte](https://pubmed.ncbi.nlm.nih.gov/?term=Nauntofte+B&cauthor_id=19709350), B., Stoltze, K. Hyposalivation, xerostomia and oral health status of HIV-infected subjects in Thailand before HAART era. J. Oral Pathol. Med. **39**(1):28-34. <https://doi.org/10.1111/j.1600-0714.2009.00826.x>. (2010).
12. Jiménez, M. M. C., Harris, R. J., Palomino, R. W., Díaz, C. A. J., Puello, R. E. (2012) Manifestaciones orales en pacientes VIH/SIDA asociadas a tratamiento antirretroviral y el estado inmunológico en dos fundaciones de la ciudad de Cartagena. Av. Odontoestomatol. **28**(4):181-189.
13. [Anaya-Saavedra](https://pubmed.ncbi.nlm.nih.gov/?term=Anaya-Saavedra+G&cauthor_id=23278731), [G](https://pubmed.ncbi.nlm.nih.gov/23278731/#full-view-affiliation-1)., [Flores-Moreno](https://pubmed.ncbi.nlm.nih.gov/?term=Flores-Moreno+B&cauthor_id=23278731), B., [García-Carrancá](https://pubmed.ncbi.nlm.nih.gov/?term=Garc%C3%ADa-Carranc%C3%A1+A&cauthor_id=23278731), A., [Irigoyen-Camacho](https://pubmed.ncbi.nlm.nih.gov/?term=Irigoyen-Camacho+E&cauthor_id=23278731), E., Guido-Jiménez, M., Ramírez-Amador, V. HPV oral lesions in HIV-infected patients: the impact of long-term HAART. J. Oral Pathol. Med. **42**(6), 443-449. <https://doi.org/10.1111/jop.12032> (2013).
14. [Camacho-Aguilar](https://pubmed.ncbi.nlm.nih.gov/?term=Camacho-Aguilar+S&cauthor_id=29480634) S, [Ramírez-Amador](https://pubmed.ncbi.nlm.nih.gov/?term=Ram%C3%ADrez-Amador+V&cauthor_id=29480634) [V](https://pubmed.ncbi.nlm.nih.gov/29480634/#full-view-affiliation-1), [Rosendo-Chalma](https://pubmed.ncbi.nlm.nih.gov/?term=Rosendo-Chalma+P&cauthor_id=29480634) P, [Guido-Jiménez](https://pubmed.ncbi.nlm.nih.gov/?term=Guido-Jim%C3%A9nez+M&cauthor_id=29480634) [M](https://pubmed.ncbi.nlm.nih.gov/29480634/#full-view-affiliation-2)[,](https://pubmed.ncbi.nlm.nih.gov/?term=Garc%C3%ADa-Carranc%C3%A1+A&cauthor_id=29480634) García-Carrancá, A., Anaya-Saavedra, G. Human papillomavirus load in benign HPV-associated oral lesions from HIV/AIDS individuals. Oral Dis. **24**(1-2), 210-214. <https://doi.org/10.1111/odi.12732> (2018).
15. [El-Mallawany](https://pubmed.ncbi.nlm.nih.gov/?term=El-Mallawany+NK&cauthor_id=27082863), N. K., et al. Clinical Factors Associated with Long-Term Complete Remission versus Poor Response to Chemotherapy in HIV-Infected Children and Adolescents with Kaposi Sarcoma Receiving Bleomycin and Vincristine: A Retrospective Observational Study. PLoS One 11(4), e0153335. <https://doi.org/10.1371/journal.pone.0153335> (2016).
16. [Yengopal](https://pubmed.ncbi.nlm.nih.gov/?term=Yengopal+V&cauthor_id=26879655), V., [Kolisa](https://pubmed.ncbi.nlm.nih.gov/?term=Kolisa+Y&cauthor_id=26879655), [Y](https://pubmed.ncbi.nlm.nih.gov/26879655/#full-view-affiliation-1)., [Thekiso](https://pubmed.ncbi.nlm.nih.gov/?term=Thekiso+MD&cauthor_id=26879655), M. D. [Molete](https://pubmed.ncbi.nlm.nih.gov/?term=Molete+MP&cauthor_id=26879655) MP. The child and adolescent with HIV in resource poor countries. Oral Dis. **22** Suppl 1, 25-34. <https://doi.org/10.1111/odi.12411> (2016).
17. [Arubaku](https://pubmed.ncbi.nlm.nih.gov/?term=Arubaku+W&cauthor_id=35689234), W., et al. Prevalence, correlates and treatment needs of dental caries among people on antiretroviral therapy in Uganda: a cross sectional study. BMC Oral Health **22**(1), 231. <https://doi.org/10.1186/s12903-022-02256-5> (2022).
18. Nittayananta, W. Oral fungi in HIV: challenges in antifungal therapies. Oral Dis. **22** Suppl 1, 107-113. <https://doi.org/10.1111/odi.12394> (2016).
19. Ricardo, J. H., Herrera, A. H. Candidiasis bucal en pacientes VIH/SIDA asociada a niveles de linfocitos T CD4 y terapia antirretroviral. Ver. Cub. Estomatol. 53, 9-14. (2016)
20. [Shintani](https://pubmed.ncbi.nlm.nih.gov/?term=Shintani+T&cauthor_id=31426660), [T](https://pubmed.ncbi.nlm.nih.gov/31426660/#full-view-affiliation-1)., et al. Oral environment and taste function of Japanese HIV-infected patients treated with antiretroviral therapy. AIDS Care, **32**(7), 829-834. <https://doi.org/10.1080/09540121.2019.1656327> (2020).
