# Supplementary material for: Oral manifestations in HIV-positive individuals under highly active antiretroviral therapy: a systematic review and meta-analysis of prevalence data
Source: BMC Oral Health. 2026 Mar 31;26:816. doi: 10.1186/s12903-026-08182-0 (PMC13159240; doi:10.1186/s12903-026-08182-0)
Supplement: Supplementary file 3 — Supplementary Material 3. [file 12903_2026_8182_MOESM3_ESM.docx]

**Supplementary Table 2.** Pooled prevalence of each oral manifestation.

| **Oral manifestation** | **n. studies (total sample)** | **Pooled prevalence % (95%CI)** | **I² % (95% CI)** |
| --- | --- | --- | --- |
| Pseudomembranous candidiasis | 15 (4299) | 7.1 (2.8; 13.0) | 96.4 (95.2; 97.3) |
| Erythematous candidiasis | 15 (4241) | 8.0 (3.9; 13.2) | 95.8 (94.4; 96.9) |
| Angular cheilitis | 14 (4561) | 3.8 (2.3; 5.5) | 72.6 (53.1; 83.9) |
| Herpes simplex | 13 (4358) | 1.2 (0.4; 2.2) | 72.3 (51.7; 84.2) |
| Kaposi's sarcoma | 12 (4365) | 1.2 (0.5; 2.2) | 71.1 (48.1; 84.0) |
| Oral hairy leukoplasia | 21 (5510) | 3.8 (1.9; 6.2) | 91.9 (88.9; 94.0) |
| Oral mucosa hyperpigmentation | 13 (2210) | 16.2 (7.7; 27.0) | 97.5 (96.7; 98.1) |
| Recurrent oral ulceration | 19 (5679) | 4.0 (1.8; 6.7) | 96.8 (95.9; 97.5) |
| Salivary gland diseases | 8 (1276) | 3.9 (1.6; 7.1) | 78.6 (57.9; 89.1) |
| Necrotizing ulcerative gingivitis | 9 (3504) | 2.3 (1.3; 3.6) | 49.3 (0.0; 76.4) |
